# Fatal Community-acquired Pneumonia in Children Caused by Re-emergent Human Adenovirus 7d Associated with Higher Severity of Illness and Fatality Rate

**DOI:** 10.1038/srep37216

**Published:** 2016-11-16

**Authors:** Zhiwu Yu, Zhiwei Zeng, Jing Zhang, Yuxian Pan, Manjun Chen, Yonghui Guo, Nan Yu, James Chodosh, Ning Fu, Xiaoyan Che, Qiwei Zhang

**Affiliations:** 1 Laboratory of Emerging Infectious Diseases and Division of Laboratory Medicine, Zhujiang Hospital, Southern Medical University, Guangzhou, Guangdong 510282, China; 2 Biosafety Level-3 Laboratory, School of Public Health, Southern Medical University (Guangdong Provincial Key Laboratory of Tropical Disease Research), Guangzhou, Guangdong 510515, China; 3 Department of Ophthalmology, Howe Laboratory, Massachusetts Eye and Ear Infirmary, Harvard Medical School, Boston, Massachusetts 02114, USA

## Abstract

Human adenoviruses (HAdVs) are highly contagious pathogens causing acute respiratory disease (ARD), such as community-acquired pneumonia. HAdV-7d, a re-emergent genomic variant, has been recently reported in Asia and the United States after a several-decade absence. However, whether HAdV-7d is associated with higher severity than other types is currently unclear. In this study, the clinical and epidemiological investigation showed that fever, cough, and sore throat were the three most common respiratory symptoms of HAdV infections. HAdV-7 caused longer duration of fever, higher morbidity of tachypnea/dyspnea, pleural effusion, diarrhea, hepatosplenomegaly, consciousness alteration, as well as higher rates of pneumonia, mechanical ventilation and higher fatality rate (28.6%) than other types, particularly HAdV-3 and HAdV-2. The genomes of seven HAdV-7d isolates from mild, severe, and fatal cases were sequenced and highly similar with each other. Surprisingly, two isolates (2011, 2012) had 100% identical genomes with an earlier strain from a fatal ARD outbreak in China (2009), which elucidates the virus origin and confirms the unexpected HAdV genomic conservation and stability. Phylogenetic analysis indicated that L1 52/55-kDa DNA packaging protein may be associated with the higher severity of illness and fatality rate of HAdV-7. Clinicians need to be aware of HAdVs in children with ARD.

Human adenoviruses (HAdVs) are highly contagious pathogens and associated with a broad spectrum of human diseases, such as acute respiratory disease (ARD), including bronchopneumonia, community-acquired pneumonia (CAP) and upper respiratory tract infection^1^,^2^,^3^,^4^. The infections in immunocompetent individuals are usually mild and self-limiting, but more and more severe and even fatal cases in children and immunocompetent adults caused by emergent and re-emergent HAdV types have been reported^1^,^3^,^5^,^6^,^7^,^8^,^9^,^10^.

Of the seven HAdV species, species B (mostly HAdV-3, -7, -14, and -55) and species E (HAdV-4) are highly associated with ARD, which account for a high proportion of respiratory diseases both in children and adults throughout the world^1^,^2^,^11^,^12^,^13^,^14^,^15^,^16^,^17^,^18^,^19^,^20^,^21^,^22^,^23^. HAdV-7 is one of the most important respiratory HAdVs and has been circulating both in military and civilian populations. It accounts for nearly 20% of all HAdVs reported to the World Health Organization^24^ and is associated with severe respiratory disease^13^,^25^,^26^,^27^.

Recently, more and more ARD outbreaks with severe or even fatal cases caused by HAdV-7 have been reported in Asia, especially in China, which has a large and high-density population^1^,^6^,^11^,^13^,^15^,^16^,^28^,^29^,^30^,^31^. The three recent isolates circulating in Southern China (DG01; 2011), Southwestern China (CQ1198; 2010), and Northwestern China (0901HZ/ShX; 2009) were identified as a re-emergent genome type 7d (HAdV-7d) after approximately 20-year absence in China^1^. In the United States, the recent emergence of HAdV-7d during 2013–2014 caused many hospitalizations and fatal CAP in adult civilians^27^,^32^. These emergent or re-emergent HAdV-7d variants may have powerful potential to cause more outbreaks, not only in Asia with its dense population centers, but also in Americas.

More and more ARD outbreaks and fatal cases caused by HAdVs have aroused increasing public health concern about the emergence and re-emergence of human adenoviruses variants (http://english.caijing.com.cn/2012-03-13/111741354.html). Vaccines against HAdV-4 and HAdV-7 have been adopted once again for military recruits (http://www.baltimoresun.com/health/bs-hs-military-adenovirus-20120312-story.html). Therefore, in this study, we investigated the clinical features and molecular epidemiology of HAdVs circulating among pediatric inpatient and outpatient children with ARD during 2010-2012 in Guangzhou, Southern China. Furthermore, in order to better understand the association between the disease severities and HAdV types, the comparison of clinical and epidemiological features as well as comparative genomic analysis of the seven HAdV-7d isolates was also performed.

## Results

### Respiratory pathogens detected in pediatric patients in Southern China, 2010–2012

**Figure 1:**
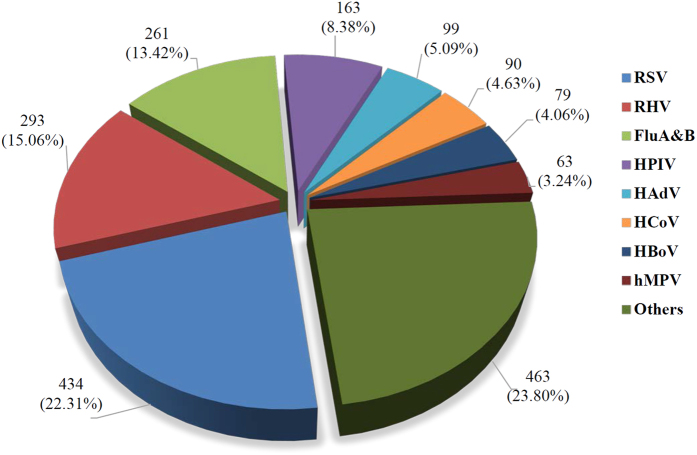
Respiratory pathogens detected in pediatric ARD patients in Southern China, 2010–2012. Human adenovirus (HAdV), influenza virus A and B (Flu A & B), human parainfluenza virus (HPIV) types 1–4, respiratory syncytial virus (RSV), respiratory enteroviruses and rhinoviruses (RHV), human metapneumovirus (hMPV), human coronaviruses (HCoV) 229E, OC43, NL63 and HKU1, human bocavirus (HBoV) were detected from 1,945 ARD cases by real-time fluorescent PCR. The number of positive cases and corresponding percent are shown for all the detected pathogens.

From January 2010 to December 2012, 1,482 of 1,945 ARD cases of inpatients and outpatients (76.2%) were positive for at least one respiratory virus, including 434 (22.31%) cases of RSV, 293 (15.06%) cases of RHV, 261 (13.42%) cases of influenza A and B, 163 (8.38%) cases of HPIV, 99 (5.09%) cases of HAdV, 90 (4.63%) cases of HCoV, 79 (4.06%) cases of HBoV, 63 (3.24%) cases of hMPV (Fig. 1). There were 463 (23.80%) cases with an undetected pathogen from the above.

### Molecular types of human adenoviruses circulating in Southern China

Seventy-nine specimens from the 99 enrolled patients were CPE-positive by cell culture. The predominant circulating types were HAdV-3 (61; 61.6%) and HAdV-7 (15; 15.2%), followed by HAdV-2 (12; 12.1%) and HAdV-1 (4; 4.1%). Thirty-four cases (34.3%) had co-infection with at least one respiratory virus. RSV and HBoV (8, 23.5% each) were the most common respiratory pathogens in children co-infected with HAdVs. HAdV-14, HAdV-21, HAdV-55 (2; 2% each), and HAdV-19 (1; 1%) were identified without co-infections with any viruses (Table 1).

### Epidemiology of human adenoviruses in children with ARD

**Figure 2:**
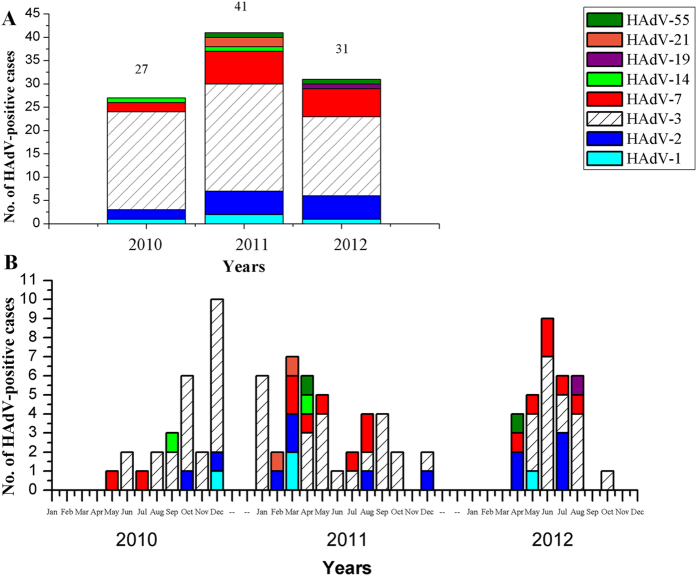
The temporal and type distribution of HAdVs associated with ARD in Guangzhou, Southern China from 2010 to 2012. There were 27 HAdV-positive cases in 2010, 41 cases in 2011 and 31 cases in 2012 (**A**). The monthly ARD cases associated with specific HAdV types were also indicated (**B**).

The temporal distribution of 99 cases of HAdVs was shown in Fig. 2. In 2010, there were 27 ARD cases caused by HAdV-1, -2, -3, -7, and -14; in 2011, there were 41 HAdV-related ARD cases. HAdV-1, -2, -3 and -7 were identified every year, whereas HAdV-14 and HAdV-55 emerged in 2010 and 2011, respectively (Fig. 2A). HAdV prevalence peaked in December 2010, March 2011, and June 2012, respectively (Fig. 2B).

The seasonal distributions of the HAdVs were further analyzed (Table 2). Overall, the prevalence peaked in the summer (33; 7.67%). There was no significant statistical difference in the prevalence peaks between the summers in 2010–2012 (6.49–9.59%, *p *= 0.763). However, when looking at the seasonal peaks in each year, HAdV-positive rate in the winter of 2010 (10; 14.71%) was significantly higher than the other winters of 2011 and 2012 (*p *< 0.001); the prevalence of pediatric ARD associated with HAdVs was significantly higher in 2010 (27; 6.51%) and 2011 (41; 6.81%) than in 2012 (31; 3.34%).

### Demographic characteristics of HAdV-positive children with ARD in 2010–2012

Overall, the median age with HAdV infection was 33.5 months (0.7 months - 13 years); 89% were under 5 years; the male-to-female ratio was 2.19:1. Of the 99 HAdV-positive cases, 69 (69.7%) were hospitalized; 24 (24.2%) children without underlying health conditions were admitted to the intensive care unit (Table 3).

### Comparison of clinical features and laboratory findings of the hospitalized children caused by HAdV-7 and other types

Nearly all the hospitalized patients associated with HAdV infections had fever (94.1%) (Table 4). The median peak of body temperature was 39.7 °C (range: 37.5–42 °C). The median duration of fever was 5.3 days (range: 1–23 days). Besides fever, sore throat (79.4%) and cough (75%) were also the most common respiratory symptoms of the inpatients. Lower respiratory tract infections were the most common diagnoses in the hospitalized patients, including 28 bronchopneumonia (41.2%), 17 pneumonia (25%), and 7 bronchitis (10.3%) cases. The median duration of hospitalization was 10.6 days (range: 2–83 days). Two inpatients infected by HAdV-3 and five patients infected by HAdV-7 required mechanical ventilation during intensive care. Four inpatients infected by HAdV-7 without any co-infection died after treatment; three deaths were associated with multiple organ failure, and the other one associated with acute respiratory distress syndrome.

Fourteen of 15 children infected with HAdV-7 were hospitalized and 8 (53.3%) needed intensive care (Table 3). Compared with ARD inpatients caused by the other types, the inpatients infected by HAdV-7 had more severe clinical symptoms (Table 4). They had longer duration of fever (8.6 vs. 4.4 days; *p *= 0.039), the higher morbidity of tachypnea/dyspnea (71.4% vs. 29.6%; *p *= 0.004), more pleural effusion (57.1% vs. 3.7%; *p *< 0.001), diarrhea (42.8% vs. 11.1%; *p *= 0.012), hepatosplenomegaly (57.1% vs. 24.1%; *p *= 0.025), and consciousness alteration (28.6% vs. 1.8%; *p *= 0.005). The clinical outcomes of these inpatients were also more severe than non-HAdV-7 infected inpatients, including the occurrence of pneumonia (57.1% vs. 16.7%; *p *= 0.004), mechanical ventilation required (35.7% vs. 3.7%; *p *= 0.003), and significantly higher fatality rate (28.6% vs. none; *p *= 0.001).

Some laboratory findings from the HAdV-7-positive inpatients were also different from those infected by other types (Table 5). HAdV-7-positive inpatients had lower white blood cell count (6.85 vs. 12.95 × 10^9^ cells/L; *p *< 0.001), platelet count (188 vs. 273 × 10^9^ cells/L; *p *= 0.007), hemoglobin levels (95.08 vs. 108.6 g/L; *p *= 0.001) and serum calcium levels (1.92 vs. 2.24 mmol/L; *p *< 0.001) than the inpatients infected by other types (Table 5). On the contrary, the lactate dehydrogenase levels (940.8 vs. 429.5 IU/L; *p *= 0.037), the morbidity of hyponatremia (36.4% vs. 23.1%; *p *= 0.022), and the albuminuria rate (45.5% vs. 4.8%; *p *= 0.003) were found significantly higher in the HAdV-7 group than the other group.

### Comparative genomic analysis of the seven HAdV-7 isolates associated with severe and mild ARD

In order to better understand the association of the genetic characteristics of HAdV-7 and clinical manifestations and outcomes between children with severe ARD and mild respiratory disease, seven representative HAdV-7 isolates, including one from an outpatient (OP) causing mild respiratory disease (OP01_2011), three from inpatients (IP; fatal outcome) named IP01_2010, IP02_2011 and IP03_2011, two from severe cases (IP04_2011 and IP05_2012) (required intensive care and mechanical ventilation), and one from an IP with comparatively mild ARD (IP06_2012) (diagnosed as bronchitis; discharged after 4-day hospitalization), were selected to perform next-generation whole-genome sequencing on Ion Torrent PGM^TM^ using 318 v2 chips; it yielded 6,063,042 reads with an average length of 182 bp, which was obtained with a mean coverage of 2,409x. The assembly in each samples yielded 3 or 4 contigs after PASA analysis. All of these samples have one long contig covering above half of the reference genome (N50 > 17 kb). The local coverage rates varied between 91–92%. The numbers of gaps identified in each samples were 3–5. These gaps were covered by Sanger sequencing for more than three times (84 in total) following PCR amplification. The complete genome sequences of strains OP01_2011, IP01_2010, IP02_2011, IP03_2011, IP04_2011, IP05_2012 and IP06_2012 were deposited in the GenBank database under accession nos. KP670857, KP670858, KP670856, KP670855, KP670861, KP670860, and KP670859, respectively.

Sequence analysis demonstrated these seven isolates had identical hexon, fiber and penton base genes. The further genome pairwise alignments indicated that the genome sequences of the 2 isolates (IP04_2011 and IP06_2012) were 100% identical with each other as well as with the previous HAdV-7 strain 0901HZ/ShX/2009, which was associated with the fatal pneumonia in an ARD outbreak in the Shaanxi Province of China in 2009^7^,^15^. The genome sequence identities between both isolates and the other five isolates were extremely high (more than 99.98%). When compared with strain 0901HZ/ShX/2009, the genomes of both isolates OP01_2011 and IP05_2012 contained only one single T insertion in E3 non-coding region and Virus-Associated (VA) RNA II, respectively (Table 6). Isolate IP01_2010 contained four nucleotide mutations (G to A) in membrane protein E3 RID-alpha, which led to four non-synonymous substitutions in this protein. There was only one synonymous substitution (C to T) in hexon assembly-associated protein of isolates IP02_2011. Isolate IP03_2011 had one more non-synonymous substitution located in 34 kDa protein (G to T).

### Genome type determination of the seven HAdV-7 isolates

**Figure 3:**
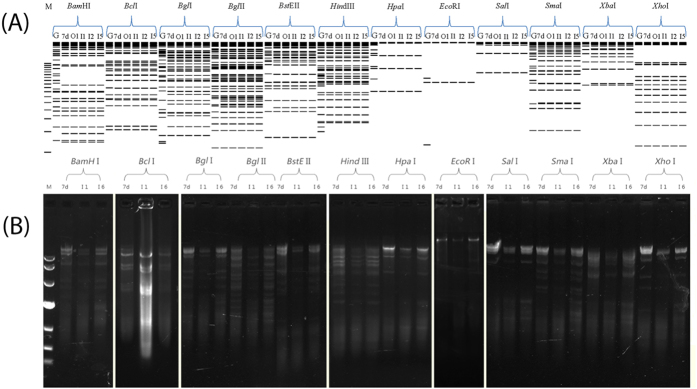
Determination of genome types of HAdV-7 isolates from children with ARD in Guangzhou, Southern China. The *in silico* restriction endonuclease analysis (REA) was performed using the Vector NTI 11.5 (Invitrogen Corp; San Diego) to identify the genome types of circulating HAdV-7 isolates OP01_2011 (lane O1; KP670857), IP01_2010 (lane I1; KP670858), IP02_2011 (lane I2; KP670856) and IP05_2012 (lane I5; KP670860) (A). Genome sequences of strains Gomen type 7 prototype Gomen (lane G; AY594255) and DG01_2011 7d (lane 7d; KC440171) were also analyzed with *Bam*HI, *Bcl*I, *Bgl*I, *Bgl*II, *Bst*EII, *Hin*dIII, *Hpa*I, *Eco*RI, *Sal*I, *Sma*I, *Xba*I, and *Xho*I (TaKaRa Corp.; China) respectively, as described earlier (Zhao *et al. Sci Rep*, 2014, 4; doi:10.1038/srep07365). The agarose gel electrophoresis of RE digested genomic DNA was also performed to confirm the *in silico* REA (**B**). IP01_2010 (lane I1; KP670858) and IP06 (lane I6; KP670859) were chosen to be analyzed. All the seven HAdV-7 isolates in this study have identical REA profiles with each other, different from the Gomen prototype, identified as genome type HAdV-7d. M, molecular weight markers corresponding to a 500 bp DNA Ladder (**A**) and DL10,000 DNA Marker (**B**) respectively. *Nota bene*: the agarose gel electrophoresis of RE digested genomic DNA was not run at the same time but under the same conditions.

Although seemingly antiquated when compared with genome sequencing, REA profiles are still helpful to compare the unsequenced but previously reported genome types and strains^1^. The genome types of all seven HAdV-7 isolates were determined by comparing REA profiles from both *in silico* (Fig. 3A) and agarose gel electrophoresis (Fig. 3B) with other HAdV-7 genome types reported earlier^1^. Isolates OP01_2011, IP01_2010, IP02_2011, and IP05_2012 were chosen as representatives for *in silico* REA (Fig. 3A); IP01_2010 and IP06_2012 were chosen for agarose gel electrophoresis REA (Fig. 3B). The REA profiles of HAdV-7 prototype strain Gomen and HAdV-7d strain DG01_2011 were chosen as references. According to the genome type denomination of Li, *et al*.^33^, all of these seven strains are identified as HAdV-7d (Fig. 3), evidenced by the REA patterns and identical with the first reported HAdV-7d^33^,^34^,^35^, as well as with the re-emergent HAdV-7d strains DG01_2011 and 0901HZ/ShX/2009^1^,^7^,^15^.

### Phylogenetic analysis of hexon, fiber, penton base genes, L1 52/55 kDa DNA packaging protein gene and the whole genomes

**Figure 4:**
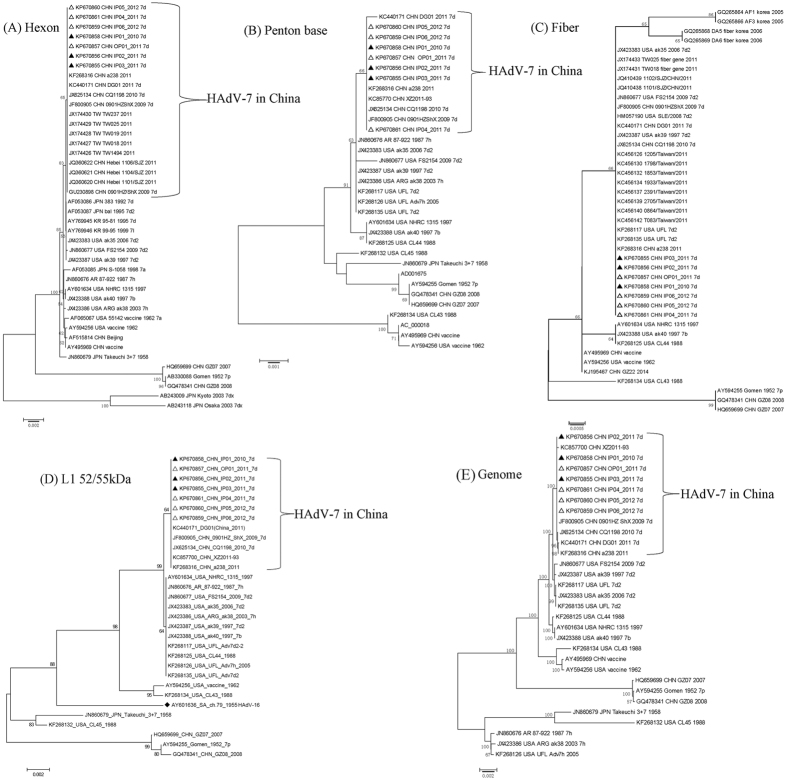
Phylogenetic analysis of the HAdV-7 isolates associated with mild, severe and fatal respiratory diseases. Panels A, B, C, D and E represent the phylogenetic trees of hexon, penton genes, fiber, L1 52/55 kDa DNA packaging protein, and the whole genomes of HAdV-7, respectively. The neighbor-joining trees with 1,000 replicates were constructed using the MEGA 6.06 software and by applying default parameters, with a maximum-composite-likelihood method. The HAdV-7d isolates with whole genomes sequenced in this study were highlighted with triangles, including the strains from fatal cases indicated in filled triangles. The archived HAdV-7 genome sequences, hexon, penton, fiber, and 52/55 kDa sequences with GenBank Accession Numbers, country of isolation, strain name, year of isolated (if available), and genome type (if available) were included in this phylogenetic analysis.

Phylogenetic analysis of the hexon, penton base, and fiber gene of China isolates showed no substantial differences from those found in the Japanese, Korean and US isolates (bootstrap values less than 80) (Fig. 4A, 4B, 4C). Phylogenetic analysis of fiber gene showed that this gene was highly conserved in all the HAdV-7 isolates analyzed (Fig. 4C). However, the phylogenetic tree based on L1 52/55 kDa DNA packaging protein indicated that the isolates circulating in China, USA, and Argentina were much closer to HAdV-16, and distinct from the HAdV-7 prototype Gomen strain (1952) (Fig. 4D). Additionally, phylogenetic analysis of 31 available HAdV-7 whole genomes revealed that the seven HAdV-7d isolates represented an unusual clade (bootstrap value: 100) along with the other HAdV-7d strains circulating in China, including 0901/HZ/ShX/2009, CQ1198_2010, DG01_2011 (Fig. 4E), which was distinct from the HAdV-7d2 USA strains and the prototype Gomen strain (Fig. 4A, 4B, 4D). The overall genome sequences of these hyper-virulent isolates are very distinct.

## Discussion

CAP is a common and serious infection that afflicts children throughout the world^36^. It is the world’s most important cause of child death^37^. In the United States, the annual incidence of pneumonia was 15.7 cases per 10,000 children, with the highest rate among children younger than 2 years of age (62.2 cases per 10,000 children)^38^. In the developing world, the annual incidence is higher and CAP is also more severe and is the largest killer of children^39^,^40^,^41^. Respiratory syncytial virus, HAdV and human metapneumovirus were more common among children younger than 5 years of age than among older children^38^. Pediatric ARD, especially CAP, caused by HAdV infection has raised the public health concerns worldwide due to its high morbidity and mortality, especially in immunocompromised population. However, few large-scale epidemiologic and clinical data nationwide is currently available.

In our study, the HAdV infection rate was 5.09% in pediatric inpatients and outpatients in Southern China, whereas it was 12%–20.1% in hospitalized children in China^42^,^43^. The type distributions of HAdV in Southern and Northern China are completely different. In Southern China, most of HAdV-positive cases were caused by HAdV-3 (61.6%), followed by HAdV-7 (15.2%), which was similar with the previous study^43^. However, HAdV-7 (46.2%) dominated in Northern China^42^. In Southern China, the peak season of HAdV infection rates was summer, with the exception of 2010. This was consistent with the study in hospitalized children in Southern China during 2012–2013^43^, but it was different from Northern China (winter)^42^.

We also found emergent HAdV-14 and HAdV-21 and re-emergent HAdV-55 in Southern China, which may have potential to cause more outbreaks in this area, similar with ARD outbreaks in Northern China^3^,^17^,^18^,^21^,^44^. Remarkably, we found HAdV-14 emerged in September 2010, one month earlier than the first HAdV-14 strain reported in China^21^. The relationship between the two strains merits further investigation. Another study in Northern China found that HAdV-55 had higher pneumonia severity index scores in adolescent and adult patients compared with those with other types^3^. However, due to the difference both in case number of each type and in the age groups, the severity of illness caused by HAdV-7 and -55 cannot be compared between their study and ours.

Moreover, a high rate of HAdV co-infections (36.8%) with other respiratory viruses was also found. The dominant pathogens co-infected with HAdV were RSV, HBoV, HPIV and HCoV, which was similar to a report from Northern China^42^. However, no statistically significant difference was found in terms of clinical manifestations and laboratory data between HAdV co-infection and HAdV single infection.

HAdV-7d was firstly identified in 1980 in Beijing and rapidly became the major genome type circulating in China through 1990^45^. In 2009 and 2011, HAdV-7d re-emerged in China after an approximately 20-year absence and caused two ARD outbreaks in children with a fatality^1^,^7^. It was also the prevalent genome type in Japan during 1987 to 1992^46^ and in Korea during two outbreaks in 1995–1996 and 2001–2002^47^, with a high fatality rate (18%) in children^48^. Recently, it emerged to cause adult severe CAP in the USA during 2013–2014^27^,^32^. In our study, HAdV-7d caused more severe clinical manifestations and adverse consequences than other types, particularly HAdV-3 and HAdV-2 (61 and 12 cases, respectively), with longer duration of fever, higher morbidity of tachypnea/dyspnea, pleural effusion, diarrhea, hepatosplenomegaly, and consciousness alteration, as well as with higher rates of pneumonia, mechanical ventilation and death. As there were relatively few cases of HAdV-14 and HAdV-55 infection in our samples, whether HAdV-7d can cause more severe clinical manifestations or adverse consequences than these latter two types is unknown. More cases will be collected for further analysis.

In our study, whole-genome sequencing was carried out on Ion Torrent PGM^TM^ using 318 v2 chips, which yielded 6,063,042 reads with an average length of approximately 182 bp as well as a mean coverage of 2,409x. Gaps and ambiguous sequences were PCR-amplified using different primers and re-sequenced by Sanger method for more than three-fold coverage. The genome ends were determined by direct end sequencing using genomic DNA as template. Given that Ion Torrent could not generate reads at long (>14-base) homopolymer tracts, and cannot predict the correct number of bases in homopolymers >8 bases long^49^, there may be a possibility of sequencing error in these regions. Additionally, the reported sequencing error rate for Ion Torrent was 1.78%. The number of error-free reads, without a single mismatch or indel, was 15.92%^49^.

We believe that genetic differences in the HAdV-7 genomes played an important role in clinical consequences of pediatric ARD with HAdV-7d causing more severe illness. Unexpectedly, we observed that the complete genome sequences of HAdV-7d isolates from mild, severe and fatal respiratory diseases were almost indistinguishable. Also surprisingly, strains IP04_2011 (severe case) and IP06_2012 (mild case) were 100% identical with each other as well as with the fatal strain (0901/HZ/ShX/2009) from the 2009 ARD outbreak^7^,^15^; the other five strains had only a few nucleotide mutations. To our knowledge, this is the first report that the complete genomes of three isolates from different periods were identical with each other, which confirms the expected degree of genomic sequence conservation and stability of HAdVs. Based on the genome sequence identity, we conclude that strains IP04_2011 and IP06_2012 in Guangzhou originated from strain 0901/HZ/ShX/2009 in Xixiang County, Shaanxi Province, although both cities are more than 1,000 kilometers apart. The unexpectedly high genome identities between isolates from mild, severe and fatal cases indicate that the difference in HAdV-7d pathogenicity may be due to host immunity. The detailed risk factors associated with adverse consequences caused by HAdV-7d merit further investigation.

Recombination analysis revealed that the genome variant 7d differs from the 1950s-era prototype strain by a lateral gene transfer, substituting the coding region for the L1 52/55 kDa DNA packaging protein from HAdV-16^1^. Our phylogenetic analysis confirmed this recombination even in HAdV-7b, 7d, 7d2, and 7 h (Fig. 4D), while not in the prototype Gomen strain. The importance of this recombination remains unknown. However, a previous study found that this protein was expressed in both the early and late stages of infection^50^. L1 52/55 kDa proteins and IVa2 interacted in infected cells and bound *in vivo* to the packaging sequence^50^,^51^. It was an essential protein absolutely required for DNA packaging and encapsidation^52^, which contributed to the adenoviral reproduction. All the emergent pathogens (7b, 7d, 7d2, and 7h) contained this particular moderate sized recombination from HAdV-B16 into the HAdV-7 genome chassis. These data provide a clue that the L1 52/55 kDa DNA packaging protein may be associated with the HAdV-7 hypervirulent phenotype, but it requires confirmation.

In conclusion, this study identified the circulating HAdV types associated with pediatric ARD in Southern China along with clinical features and the whole-genome sequencing analysis. In Southern China, the predominant types in children were HAdV-3 and HAdV-7. The peak season of HAdV infection in 2011–2012 was summer. The re-emergent HAdV-7d isolates originated from the earlier strain associated with a fatal ARD outbreak (2009). HAdV-7d caused more severe illness and higher fatality rate in pediatric inpatients than other types. The L1 52/55 kDa DNA packaging protein may be associated with the HAdV-7 hypervirulent phenotype. Fever, cough, and sore throat were the three most common respiratory symptoms of severe HAdV infections. The vaccine against HAdV-7 is in urgent need in both China and other population-dense countries. HAdV-7d associated with higher severity of illness has re-emerged in China and may have the potential to cause more outbreaks. Clinicians should pay particular attention to HAdVs in children with ARD. We call attention to urge extensive and continuous epidemiological surveillance and genetic characterization of the currently circulating HAdV strains in China, as well as in other countries with close-quartered and/or vulnerable populations.

## Methods

### Study design and clinical specimens

This study was carried out in the pediatric department of Zhujiang Hospital, Southern Medical University. The pediatric outpatient and inpatient cases under 14 years with influenza-like symptoms and clinically confirmed ARD from 2010 to 2012 were included in this study. Nasopharyngeal swab specimens from these patients were collected. All the experimental protocols in this study were approved by the Medical Ethics Board of Southern Medical University and were carried out in accordance with the approved guidelines. The informed consents for participation in this study were obtained from guardians of all the under-aged participants. Data records of the samples, sample collection and analysis are de-identified and completely anonymous.

### Microbiological diagnostic tests

Human adenovirus (HAdV), influenza virus A and B (Flu A & B), human parainfluenza virus (HPIV) types 1–4, respiratory syncytial virus (RSV), respiratory enteroviruses and rhinoviruses (RHV), human metapneumovirus (hMPV), human coronaviruses (HCoV) 229E, OC43, NL63 and HKU1, human bocavirus (HBoV) were detected from the specimens by real-time fluorescent PCR according to the methods described earlier^53^,^54^,^55^,^56^.

### Virus culture, identification, and molecular typing of HAdVs

The nasopharyngeal swab specimens from HAdV-positive patients were inoculated into HEp-2 cells and cytopathic effect (CPE) was monitored for ten days. The HAdVs were molecular typed by PCR amplification and sequencing of all seven hypervariable regions in the hexon gene^57^. Molecular types were determined by BLASTN of the assembling contigs against existing GenBank sequences.

### HAdV-7 whole-genome sequencing, annotation and phylogenetic analysis

Purified HAdV genomic DNA was submitted to Sagene Bio Co. (Guangzhou, China) for next generation whole-genome sequencing on Ion Torrent Personal Genome Machine (PGM^TM^). In brief, genomic libraries were prepared using the Ion Xpress plus fragment library kit (Life Technologies) and barcoded adapters were linked to DNA fragments, then amplified by emulsion PCR with Ion PGM 200 Xpress Template kit (Life Technologies). After breaking the emulsion, and adding the sequencing primer and polymerase, the completely prepared Ion Sphere Particle (ISP) beads of seven samples were loaded into Ion 318 v2 chips and individually sequenced on Ion Torrent PGM, with 500 flows generating 6,063,042 reads with an average length of 182 bp, and a mean coverage of 2,409x. All the sequencing ladders were assembled with the Program to Assemble Spliced Alignments (PASA) tool^58^ and SEQMAN software from the Lasergene package (DNAStar; Madison, WI). HAdV-7 strain 0901HZ/ShX/CHN/2009 (35239 bps) was chosen as the reference genome. The PASA assembly command line is as followed: “%../scripts/Launch_PASA_pipeline.pl -c alignAssembly.config -C -R -g genome_sample.fasta –t all_sample.fasta.clean -T -u all_sample.fasta -f FL_acs.txt -ALIGNER blat gmap -CPU 2”. The configuration file was input after “-c”; “genome_sample.fasta” represented the reference genome file name. Because we used PASA to assemble virus genomes with a reference genome, “-t” and “-u” parameters followed the same fasta file of virus sequencing data, despite different file names. “-ALIGNER” parameter followed two alignment methods, blat and gmap. The other parameters were set as default. Two CPUs were used to run the program simultaneously. Gaps and ambiguous sequences were PCR-amplified using different primers and re-sequenced by Sanger method for more than three-fold coverage. Both 5′- and 3′-ends including inverted terminal repeats were sequenced directly using genomic DNA as template, as described earlier^1^. The genome sequences were annotated based on the previous annotation of HAdV-7 prototype strain (Gomen; 1952)^59^ and deposited into GenBank database. The Molecular Evolutionary Genetics Analysis (MEGA) version 6.06 software (http://www.megasoftware.net/megamac.php) was used for phylogenetic analyses of HAdV-7 hexon, fiber, penton genes, L1 52/55 kDa DNA packaging protein gene and the whole genomes, with additional sequences retrieved from GenBank database. Neighbor-joining phylogenetic trees with 1,000 boot-strap replicates were constructed using a maximum-composite-likelihood method with default parameters.

### Genome type identification by *in silico* and agarose gel electrophoresis restriction endonuclease analysis (REA)

Vector NTI Advance 11.5 (Invitrogen Corp.; San Diego, CA. USA) was used for the *in silico* REA analysis of the HAdV-7 whole-genome sequences, as described earlier^1^,^60^. Twelve restriction enzymes (*Bam*HI, *Bcl*I, *Bgl*I, *Bgl*II, *Bst*EII, *Eco*RI, *Hin*dIII, *Hpa*I, *Sma*I, *Sal*I, *Xba*I, and *Xho*I) (TaKaRa Corp.; China) were chosen according to the nomenclature system developed by Li and colleagues^45^. Genome types of HAdV-7 were determined by comparing both *in silico* and agarose gel electrophoresis REA profiles with other HAdV-7 genome types reported earlier^1^.

### Statistical analysis

Data statistical operations were performed by the SPSS program. Descriptive statistics was used for all variables; the continuous variables were summarized as medians and ranges, and the categorical variables were summarized as frequencies and proportions. Student’s *t*-test, Chi square test or Fisher exact test was used where appropriate, to determine the difference between groups.

### GenBank accession numbers

The sequences used for phylogeny analyses are summarized in Supplementary Table S1, with GenBank accession number, country of isolation, strain name, year of isolated (if available), and genome type (if available) included.

## Additional Information

**How to cite this article**: Yu, Z. *et al*. Fatal Community-acquired Pneumonia in Children Caused by Re-emergent Human Adenovirus 7d Associated with Higher Severity of Illness and Fatality Rate. *Sci. Rep.* **6**, 37216; doi: 10.1038/srep37216 (2016).

**Publisher’s note:** Springer Nature remains neutral with regard to jurisdictional claims in published maps and institutional affiliations.

## Supplementary Material

Supplementary Information

## Figures and Tables

**Table 1:** HAdV types and co-infections with other respiratory viruses in children with ARD in Southern China, 2010–2012.

Virus	HAdV (%)	RSV	HBoV	RHV	HPIV	hMPV	HCoV	Flu A
HAdV-3	61 (61.6)	4	6	3	5	2	5	1
HAdV-2	12 (12.1)	0	1	1	1	1	1	
HAdV-7	15 (15.2)	3	1	1				
HAdV-1	4 (4.1)	1						
HAdV-14	2 (2)							
HAdV-19	1 (1)							
HAdV-21	2 (2)							
HAdV-55	2 (2)							
Total (%)	99	8 (23.5)	8 (23.5)	5 (14.7)	6 (17.6)	3 (8.8)	6 (17.6)	1 (2.9)

HAdV, human adenovirus; RSV, respiratory syncytial virus; ARD, acute respiratory disease; HBoV, human bocavirus; RHV, respiratory enteroviruses and rhinoviruses; hMPV, human metapneumovirus; HPIV, human parainfluenza viruses (type 2 and 3); HCoV, human coronaviruses (OC43, NL63); Flu A, influenza A.

**Table 2:** The seasonal distribution of HAdV-positive cases in children with ARD during 2010–2012.

Year	Spring (March–May)	Summer (June–August)	Autumn (September–November)	Winter (December–February)	All	*p* value
2010	1/14 (7.14%)	5/77 (6.49%)	11/256 (4.3%)	10/68 (14.71%)	27/415 (6.51%)	0.026*
2011	18/214 (8.41%)	7/73 (9.59%)	6/111 (5.41%)	10/204 (4.9%)	41/602 (6.81%)	0.352
2012	9/281 (3.2%)	21/280 (7.5%)	1/125 (0.8%)	0/242	31/928 (3.34%)	<0.001*
Total	28/509 (5.5%)	33/430 (7.67%)	18/492 (3.66%)	20/514 (3.89%)	99/1945 (5.09%)	0.021*
*p* value	0.028*	0.763	0.125	<0.001*	0.004*	

The percent are the ratios of HAdV-positive cases to all ARD cases in the corresponding season. “^*^” indicates a significant difference between seasons or years.

**Table 3:** Demographic characteristics of 99 children with ARD according to HAdV type in Guangzhou, Southern China 2010–2012.

Characteristics	All	HAdV-1	HAdV-2	HAdV-3	HAdV-7	HAdV-14	HAdV-19	HAdV-21	HAdV-55
No. of children	99	4	12	61	15	2	1	2	2
Hospitalized	69 (69.7%)	2 (50%)	8 (66.7%)	39 (63.9%)	14 (93.3%)	2 (100%)	1 (100%)	1 (50%)	2 (100%)
Age range (m)	0.7–156	12–36	0.7–52.7	3.9–156	5.9–91.6	22.5–26.1	11	12.6–144	11.8–40.2
Median age (m)	33.5	19.9	24.4	38.2	23.2	24.3	11	78.3	26
Gender (M/F)	68/31	3/1	7/5	44/17	9/6	1/1	1/0	1/1	2/0
Intensive care	24 (24.2%)	0	0	13 (21.3%)	8 (53.3%)	1 (50%)	0	1 (50%)	1 (50%)

**Table 4:** Comparison of clinical characteristics of 68 hospitalized children infected by HAdV-7 and the other types.

Characteristics	All (n = 68)	HAdV-7 (n = 14)	The other types (n = 54)	*p* value
Fever	64 (94.1)	14 (100)	50 (92.6)	0.574
Duration days (range)*	5.3 (1–23)	8.6 (3–23)	4.4 (1–21)	0.039
Median peak temperature (range)	39.7 (37.5–42)	39.6 (38.4–40.6)	39.8 (37.5–42)	0.555
Cough	51 (75)	13 (92.8)	38 (70.4)	0.162
Runny nose	21 (30.9)	4 (28.6)	17 (31.5)	1.000
Sore throat	54 (79.4)	12 (85.7)	42 (77.8)	0.717
Expectoration	28 (41.2)	8 (57.1)	20 (37.0)	0.173
Rales	28 (41.2)	8 (57.1)	20 (37.0)	0.173
Wheezing	18 (26.5)	6 (42.8)	12 (22.2)	0.173
Tachypnea/dyspnea*	26 (38.2)	10 (71.4)	16 (29.6)	0.004
Pleural effusion*	10 (14.7)	8 (57.1)	2 (3.7)	<0.001
Cyanosis	5 (7.3)	2 (14.3)	3 (5.6)	0.272
Tonsillitis	33 (48.5)	5 (35.7)	28 (51.8)	0.282
Extrapulmonary manifestations				
Skin rash	8 (11.8)	1 (7.1)	7 (12.9)	1.000
Conjunctivitis	4 (5.9)	1 (7.1)	3 (5.6)	1.000
Diarrhea*	12 (17.6)	6 (42.8)	6 (11.1)	0.012
Vomiting	9 (13.2)	3 (21.4)	6 (11.1)	0.377
Hepatosplenomegaly*	21 (30.9)	8 (57.1)	13 (24.1)	0.025
Seizure	8 (11.8)	2 (14.3)	6 (11.1)	0.665
Consciousness alteration*	5 (7.3)	4 (28.6)	1 (1.8)	0.005
Clinical outcomes				
Upper respiratory tract infection	16 (23.5)	1 (7.1)	15 (27.8)	0.161
Bronchitis	7 (10.3)	2 (14.3)	5 (9.3)	0.627
Bronchopneumonia	28 (41.2)	3 (21.4)	25 (46.3)	0.092
Pneumonia*	17 (25)	8 (57.1)	9 (16.7)	0.004
Median duration of hospitalized days (range)	10.6 (2–83)	18.7 (3–83)	8.5 (2–45)	0.075
Mechanical ventilation*	7 (10.3)	5 (35.7)	2 (3.7)	0.003
Death*	4 (5.9)	4 (28.6)	0	0.001

The values indicate the number (%) of corresponding children. “HAdV-7” group are the children infected by HAdV-7; “The other type” group are the children infected by adenovirus types 1, 2, 3, 14, 19, 21 and 55. “^*^”: a significant difference between groups.

**Table 5:** Comparison of laboratory findings of 68 hospitalized children with ARD associated with HAdV-7 and the other types.

Laboratory data and no. patients tested	All	HAdV-7 (n = 14)	The other types (n = 54)	*p* value
*WBC, ×10^9^ cells/L, n = 68	11.69	6.85 (1.9–13.7)	12.95 (4–34.84)	<0.001
LYM, ×10^9^ cells/L, n = 68	3.1	2.65 (0.52–4.84)	3.23 (0.47–20.67)	0.477
*Platelets, ×10^9^ cells/L, n = 68	256	188 (36–377)	273 (32.3–595)	0.007
*Hemoglobin, g/L, n = 68	105.8	95.08 (69–115)	108.6 (67–135)	0.001
CRP, mg/L, n = 63	29.03	39.2 (1.05–169)	26.12 (0.16–159)	0.248
AST, IU/L, n = 66	87.8	156.4 (29–629)	69.3 (15–1460)	0.143
ALT, IU/L, n = 64	26.2	28.1 (13–102)	25.7 (9–268)	0.813
*LDH, IU/L, n = 62	528.4	940.8 (258–2454)	429.5 (151–2020)	0.037
Creatinine, μmol/L, n = 66	43.5	41.14 (29–65)	44.17 (12–104)	0.486
Urea, mmol/L, n = 66	2.99	2.6 (0.7–7.1)	3.1 (0.6–8.4)	0.29
Sodium, mmol/L (range), n = 66	136.8	135.4 (129.1–140.7)	137.1 (130.3–145.2)	0.082
*Hyponatremia, no. (%) patients	20 (30.3%)	8 (36.4%), n = 14	12 (23.1%), n = 52	0.022
*Calcium, mmol/L, n = 63	2.18	1.92 (1.55–2.43)	2.24 (1.86–2.64)	<0.001
*Albuminuria, no. (%) patients, n = 53	7 (13.2%)	5 (45.5%), n = 11	2 (4.8%), n = 42	0.003
Hematuria, no. (%) patients, n = 53	5 (9.4%)	3 (27.3%), n = 11	2 (4.8%), n = 42	0.054
Urinary casts, no. (%) patients, n = 53	3 (5.6%)	2 (18.2%), n = 11	1 (2.4%), n = 42	0.106

The values are median (range) of tested number. WBC, white blood cell; LYM, lymphocyte; PLT, platelet; Hb, hemoglobin; CRP, C-reactive protein; AST, aspartate aminotransferase; ALT, alanine aminotransferase; LDH, lactate dehydrogenase; hyponatremia, serum sodium <135 mmol/L. “^*^”: a significant difference between groups.

**Table 6:** Comparative genomic analysis of the seven HAdV-7d isolates from this study.

Region	Product	Position	Mutation in DNA							Amino acid substitution						
			OP01	IP01	IP02	IP03	IP04	IP05	IP06	OP01	IP01	IP02	IP03	IP04	IP05	IP06
**L1**	Virus-associated RNA II	10815	—	—	—	—	—	Insertion of T	—	—	—	—	—	—	—	—
**L4**	Hexon assembly- associated protein	24298	—	—	C → T	C → T	—	—	—	—	—	A → A	A → A	—	—	—
**E3**	Non-coding region	29947	Insertion of T		—	—	—	—	—	—	—	—	—	—	—	—
**E3**	membrane protein E3 RID-alpha	29983	—	G → A	—	—	—	—	—	—	R → K	—	—	—	—	—
		30060	—	G → A	—	—	—	—	—	—	D → N	—	—	—	—	—
		30162	—	G → A	—	—	—	—	—	—	D → N	—	—	—	—	—
		30178	—	G → A	—	—	—	—	—	—	R → Q	—	—	—	—	—
**E4**	34 kDa protein	33155	—	—	—	G → T	—	—	—	—	—	—	P → Q	—	—	—

The reference genome is HAdV-7 strain 0901HZ/ShX/2009. Nucleic acid and amino acid sequence mutations are noted, along with their genome locations of the coding regions. “—”: no change or not applicable. Seven isolates are included: one from outpatient (OP) causing mild respiratory disease named OP01_2011, three from inpatients (IP; fatal outcome) named IP01_2010, IP02_2011 and IP03_2011, two from severe cases (required intensive care and mechanical ventilation) named IP04_2011 and IP05_2012, and one from comparatively mild respiratory disease (diagnosed with bronchitis and discharged after 4-day hospitalization) named IP06_2012.
