# Supplementary material for: Fatal Community-acquired Pneumonia in Children Caused by Re-emergent Human Adenovirus 7d Associated with Higher Severity of Illness and Fatality Rate
Source: Sci Rep. 2016 Nov 16;6:37216. doi: 10.1038/srep37216 (PMC5110970; doi:10.1038/srep37216)
Supplement: Supplementary Information [file srep37216-s1.doc]

# Supplemental materials

# Fatal Community-acquired Pneumonia in Children Caused by Re-emergent Human Adenovirus 7d Associated with Higher Severity of Illness and Fatality Rate

Zhiwu Yu^1^, Zhiwei Zeng^2^, Jing Zhang^2^, Yuxian Pan^1^, Manjun Chen^1^, Yonghui Guo^1^, Nan Yu^1^, James Chodosh^3^, Ning Fu^1^, Xiaoyan Che^1†^ & Qiwei Zhang^2,3,*^

**Supplementary Table S1.**

**The hexon, fiber, penton base, L1 52/55 kDa DNA packaging protein genes and the genome sequences of HAdV-7 available in GenBank and used for phylogeny analyses. GenBank accession number, country of isolation, strain name, year of isolated (if available), and genome type (if available) are included.**

| **Type** | **Strain** | **Year isolated** | **Country** | **Sequence Available** | **GenBank Accession Nos.** |
| --- | --- | --- | --- | --- | --- |
| HAdV-7p | Gomen | 1952 | USA | Genome  L1 52/55 kDa DNA packing protein | AY594255 |
| HAdV-7h | 87-922 | 1987 | Argentina |  | JN860676 |
|  | ARG ak38 | 2003 | USA |  | JX423386 |
|  | UFL | 2005 | USA |  | KF268126 |
|  | Takeuchi | 1958 | Japan |  | JN860679 |
|  | CL_45 | 1988 | USA |  | KF268132 |
| HAdV-7b | NHRC 1315 | 1997 | USA |  | AY601634 |
|  | CL_44 | 1988 |  |  | KF268125 |
|  | ak40 | 1997 |  |  | JX423388 |
| HAdV-7d2 | Ak35 | 2006 | USA |  | JX423383 |
|  | UFL-2 | unknown |  |  | KF268117 |
|  | UFL-3 | unknown |  |  | KF268135 |
|  | Ak39 | 1997 |  |  | JX423387 |
|  | FS2154 | 2009 |  |  | JN860677 |
|  | bal | 1995 | Japan | Hexon | AF053087 |
|  | SLE | 2008 | USA | Fiber | HM057190 |
| HAdV-7d | a238 | 2011 | China | Genome  L1 52/55 kDa DNA packing protein | KF268316 |
|  | XZ2011-93 | 2012 |  |  | KC857700 |
|  | DG01 | 2011 |  |  | KC440171 |
|  | CQ1198 | 2010 |  |  | JX625134 |
|  | 0901HZ/ShX | 2009 |  |  | JF800905 |
|  | IP_03 | 2011 |  |  | KP670855 |
|  | IP_02 | 2011 |  |  | KP670856 |
|  | OP_01 | 2011 |  |  | KP670857 |
|  | IP_01 | 2010 |  |  | KP670858 |
|  | IP_06 | 2012 |  |  | KP670859 |
|  | IP_05 | 2012 |  |  | KP670860 |
|  | IP_04 | 2011 |  |  | KP670861 |
|  | 383 | 1992 | Japan | Hexon | AF053086 |
|  | 95-81 | 1995 | Korea |  | AY769945 |
| HAdV-7l | 99-95 | 1999 | Korea | Hexon | AY769946 |
| HAdV-7a | S-1058 | 1998 | Japan |  | AF053085 |
|  | 55142 | 1962 | USA |  | AF065067 |
| HAdV-7dx | Osaka | 2003 | Japan |  | AB243118 |
|  | Kyoto | 2003 | Japan |  | AB243009 |
| HAdV-7 | GZ07 | 2007 | China | Genome  L1 52/55 kDa DNA packing protein | HQ659699 |
|  | GZ08 | 2008 | China |  | GQ478341 |
|  | Vaccine | unknown | China |  | AY495969 |
|  | Vaccine | 1962 | USA |  | AY594256 |
|  | CL_43 | 1988 | USA |  | KF268134 |
|  | TW237 | 2011 | Taiwan | Hexon | JX174430 |
|  | TW025 |  |  | Hexon  Fiber | JX174429  JX174433 |
|  | TW019 |  |  | Hexon | JX174428 |
|  | TW018 |  |  | Hexon  Fiber | JX174427  JX174431 |
|  | TW1494 |  |  | Hexon | JX174426 |
|  | 1106/SJZ |  | China | Hexon | JQ360622 |
|  | 1104/SJZ |  |  |  | JQ360621 |
|  | 1101/SJZ |  |  |  | JQ360620 |
|  | AF1 | 2005 | Korea | Fiber | GQ265864 |
|  | AF3 | 2005 |  |  | GQ265866 |
|  | DA5 | 2006 |  |  | GQ265868 |
|  | DA6 | 2006 |  |  | GQ265869 |
|  | 1101/SJZ | 2011 | China | Fiber | JQ410438 |
|  | 1102/SJZ |  |  |  | JQ410439 |
|  | GZ22 | 2014 |  |  | KJ195467 |
|  | 1205 | 2011 | Taiwan | Fiber | KC456126 |
|  | 1798 |  |  |  | KC456130 |
|  | 1853 |  |  |  | KC456132 |
|  | 1933 |  |  |  | KC456134 |
|  | 2391 |  |  |  | KC456137 |
|  | 2705 | 2011 | Taiwan | Fiber | KC456139 |
|  | 0864 |  |  |  | KC456140 |
|  | T083 |  |  |  | KC456142 |
